# Association between Hypoalbuminaemia and Mortality in Patients with Community-Acquired Bacteraemia Is Primarily Related to Acute Disorders

**DOI:** 10.1371/journal.pone.0160466

**Published:** 2016-09-09

**Authors:** Bjarne Magnussen, Kim Oren Gradel, Thøger Gorm Jensen, Hans Jørn Kolmos, Court Pedersen, Pernille Just Vinholt, Annmarie Touborg Lassen

**Affiliations:** 1 Center for Clinical Epidemiology, South, Odense University Hospital, Sdr. Boulevard 29, entrance 101, 4th floor, 5000, Odense C, Denmark; 2 Research Unit of Clinical Epidemiology, Institute of Clinical Research, University of Southern Denmark, Sdr. Boulevard 29, entrance 101, 4th floor, 5000, Odense C, Denmark; 3 Department of Clinical Microbiology, Odense University Hospital, J.B. Winsloews Vej 21, 2nd floor, 5000, Odense C, Denmark; 4 Department of Infectious Diseases, Odense University Hospital, Sdr. Boulevard 29, entrance 20, 5000, Odense C, Denmark; 5 Department of Clinical Biochemistry and Pharmacology, Odense University Hospital, Sdr. Boulevard 29, entrance 40, 5000, Odense C, Denmark; 6 Department of Emergency Medicine, Odense University Hospital, Kløvervænget 25, entrance 63-65, 5000, Odense C, Denmark; University of Leicester, UNITED KINGDOM

## Abstract

We sought to investigate whether hypoalbuminaemia was mainly caused by acute or chronic factors in patients with community-acquired bacteraemia. In this population-based study, we considered 1844 adult cases of community-acquired bacteraemia that occurred in Funen, Denmark between 2000 and 2008. We used a stepwise prognostic predisposition-insult-response-organ dysfunction (PIRO) logistic regression model by initially including age and comorbidity, then added bacterial species, and finally sepsis severity. The models were furthermore analysed using receiver operating characteristic (ROC) curves. Outcomes comprised mortality incidence on days 0–30 and 31–365 after the bacteraemia episode. Each step was performed with and without baseline albumin level measured on the date of bacteraemia. In 422 patients, their latest albumin measurement taken 8–30 days before the date of bacteraemia was also used in the analysis together with the baseline albumin level. For each decrease of 1g/L in plasma albumin level, the odds ratios (95% confidence intervals) of mortality in the period of 0–30 days after bacteraemia were 0.86 (0.84–0.88) in both predisposition (P) and predisposition-insult (PI) models and 0.87 (0.85–0.89) in the full PIRO-model. The AUC values were 0.78 and 0.66 for mortality in the period of 0–30 days in the model comprising only predisposition factors with and without albumin levels added as a factor, respectively. The AUC values in the full PIRO-model were 0.81 and 0.73 with and without consideration of albumin levels, respectively. A higher proportion of patients died within 30 days if there was a decrease in the albumin level between days 8 and 30 before bacteraemia and the actual bacteraemia date. A single plasma albumin measurement on the bacteraemia date was a better prognostic predictor of short-term mortality than the sepsis severity score.

## Introduction

Bacteraemia is most common among elderly patients with chronic comorbid conditions. It may cause severe sepsis with circulatory disturbances and a compensatory expansion of the plasma volume and extravasation. In general, it is difficult to assess to what extent a poor outcome in bacteraemic patients is related to the acute sepsis episode itself or to chronic predisposition factors often encountered in this patient cohort. Hypoalbuminaemia has traditionally been related to chronic conditions such as liver failure, malnutrition, or protein-losing enteropathy [1,2]. However, numerous studies of critically ill patients have indicated that plasma albumin (PA) in this population is a negative sign of the acute phase of the pathology and hence, an inflammation indicator, rather than a nutritional state marker [3–6]. Three processes determine the PA level: synthesis and secretion from the liver, catabolism, and distribution between the vascular system and the interstitial space [1,4,7]. The individual impact of each of these processes on the PA level, however, is difficult to quantify in real-life situations. The knowledge of disease-related changes in PA level in humans is hampered by the fact that the vast majority of observational studies only comprise a single albumin measurement [8].

In this study, we considered adult cases of community-acquired bacteraemia in order to elucidate if there was a clear association between hypoalbuminaemia and short-term (0–30 days) or long-term (31–365 days) mortality after bacteraemia, and whether this possible association was primarily caused by acute or chronic factors.

## Materials and Methods

### Study population

This study population has been described previously [9]. In brief, it included 2785 adult patients (>14 years) from the Funen County, Denmark, with an incident community-acquired bacteraemia between 2000 and 2008. We included 1844 patients who had a PA measurement on the bacteraemia date, which was defined as the date the blood culture was drawn.

### Microbiological data

All blood cultures were submitted to the Department of Clinical Microbiology at the Odense University Hospital, where they were incubated and screened for growth of microorganisms for six days or until detected positive by the Difco ESP blood culture system (Difco Laboratories, Detroit, MI, USA) in the year 2000 and the Bactec 9240 system (Becton Dickinson, Franklin Lakes, NJ, USA) in the years 2001–2008. Identification of microorganisms was based on conventional approaches [10], the Danish reference program (www.dskm.dk), and automated identification using Vitek 2 (bioMérieux, Marcy l’Etoile, France).

### Albumin level measurements

Albumin levels were measured with a Modular P analyser (Roche, Mannheim, Germany) by the bromocresol green dye-binding method.

### Community-acquired bacteremia

We defined the presence of a positive blood culture as evidence of bacteraemia based on clinical and microbiological criteria [11]. We included all infection episodes, which were associated with the presence of *Staphylococcus* spp., *Streptococcus* spp., *Enterococcus* spp., *Pseudomonas aeruginosa*, or *Enterobacteriaceae* in the blood. Coagulase-negative staphylococci or non-haemolytic streptococci found in only one bottle were considered contamination and these cases were not included in the analysis.

Bacteraemia was defined as community-acquired if the bacteraemia date was within 3 days after admission and there had been no inpatient contact within 7 days up to the bacteraemia date.

### Sepsis severity

We looked through the patients’ medical records to retrieve systemic inflammatory response syndrome (SIRS) parameters [12] as well as descriptions of any central nervous system (CNS) signs on the admission date. We defined sepsis as the condition when two or more of the three SIRS parameters were present (temperature >38°C or < 36°C; heart rate > 90 beats/min; WBC > 12,000 cells/mm^3^, or WBC < 4,000 cells/mm^3^, or when over 10% WBC were represented by immature forms). The medical records had inconsistent information on the respiratory rate or PaCO_2_ and consequently, this SIRS parameter was ignored. Sepsis was further categorized as severe sepsis/septic shock if organ dysfunction or hypotension (systolic blood pressure <90 mm Hg) occurred. Because it was not possible to assess the validity of data on the initial fluid resuscitation, we did not distinguish between severe sepsis and septic shock. We defined organ dysfunction (CNS, kidney, liver, perfusion, or lung) according to criteria described earlier [9]. Using the above-mentioned criteria, we divided the patients into the following sepsis categories: no sepsis, possible sepsis (because of missing data), sepsis, severe sepsis/septic shock, and organ dysfunction without sepsis.

### Linkage to other registries

We linked the study database to the Danish National Patient Registry [13] to retrieve information on the first-time occurrence of comorbidity between 1994 and the bacteraemia date. We used the Charlson comorbidity index that comprises 19 major comorbidity groups with higher scores being assigned to prognostically more severe diseases (e.g., diabetes mellitus, malignancy and cardiovascular diseases) [14].

Data on mortality were obtained from the Danish Civil Registration System, which contains records on the health status of all Danish residents as well as dates of death or emigration, if relevant [15].

### Statistical analysis

As the PA levels were normally distributed (data not shown), they were compared between groups defined by population characteristics (gender, age groups, Charlson comorbidity, bacterial groups, and sepsis severity) using the one-way ANOVA test. If ANOVA result was significant, the Bonferroni *post hoc* test was used for pairwise group comparisons. For ordered groups, we used the non-parametric Cuzick’s method for trend across these groups [16].

For each of the five sepsis severity groups, we computed Kaplan-Meier survival curves that represented PA level quintiles.

We used the prognostic predisposition-insult-response-organ dysfunction (PIRO) logistic regression model that comprised variables related to the predisposition (P), insult/infection (I), and response/organ dysfunction (RO) [17]. This model included the predisposition variables”gender”, “age”, “Charlson comorbidity index” (0, 1, and ≥2 points), the insult variable “main bacterial groups” (gram-positive, gram-negative, and others), and the response/organ dysfunction variable”sepsis severity group” (no sepsis, possible sepsis, sepsis, severe sepsis/septic shock, and organ dysfunction without sepsis).

We initially showed that the PA level could be used as a continuous variable in a logistic regression model with mortality incidence on days 0–30 and 31–365 after bacteraemia as outcomes, using spline, quintile, and fractional polynomial curves as reported before [18]. To facilitate reporting of the results, we subsequently divided the PA level into quintiles.

Logistic regression was used to compute odds ratios (ORs) with 95% confidence intervals (CIs) for mortality after short (0–30 days) and long periods (31–365 days) following bacteraemia. We applied a stepwise approach, in which the first step only included predisposition variables, then the insult variable was added, and finally the response/organ dysfunction variable was incorporated in the full model. These steps were performed with and without the incorporation of the PA level to assess its impact on the prediction of mortality within short or longer post-bacteraemia periods. For each of the stepwise models, we computed receiver operating characteristic (ROC) curves and values of the area under these curves (AUC) with 95% CIs. We reiterated all analyses using Cox proportional hazards regression analysis with hazard ratios (HRs) and 95% CIs. The HRs (95% CIs) did not deviate materially from the ORs (data not shown), which was expected as only 1 patient was censored within 365 days. Moreover, as HRs do not enable subsequent computation of AUCs, we report ORs in this study.

In order to determine whether hypoalbuminaemia had mainly occurred before or was caused by the bacteraemic episode, we selected patients with PA measured 8–30 days before the bacteraemia date, i.e., during the period when the patients were believed not to experience an acute phase response. We retrieved the latest PA level measurements within that period and plotted them against respective PA levels on the bacteraemia date (the baseline PA level). In this scatterplot, we further indicated whether the individual patients were alive or dead within 30 days after the bacteraemia date.

Data were considered to be significantly different if p-value < 0.05 in relevant tests. The Stata software (v.13.1, StataCorp, TX, USA) was used for all analyses.

### Ethical considerations

This study was approved by the Danish Data Protection Agency (no. 2013-41-2579). According to Danish legislation, approval by an ethics committee or consent from participants (including next of kin/caregiver in the case of children) are not required for registry-based research. Data were not anonymised prior to analysis.

## Results

### Baseline characteristics

Patients were generally old and had comorbidities. In this study cohort, 53% had severe sepsis or septic shock (Table 1).

**Table 1:** Baseline characteristics of the study cohort (n = 1844).

Population characteristics	Number (%)
**Age, years**	
Median (25^th^, 75^th^ percentile)	70.5 (57.1, 79.5)
Mean (minimum, maximum)	67.4 (15.5, 101.7)
**Females**	817 (44.3)
**Charlson comorbidity index**	
0 points	417 (22.6)
1 point	300 (16.3)
>1 points	1127 (61.1)
**Smoking**	
Never	389 (21.1)
Present	345 (18.7)
Former	190 (10.3)
Unknown	920 (49.9)
**Alcohol abuse**	
Never	698 (37.9)
Present	130 (7.0)
Former	60 (3.3)
Unknown	956 (51.8)
**Bacteria**	
Monomicrobial Gram-positive	
*Staphylococcus aureus*	273 (14.8)
*Streptococcus pneumonia*	257 (13.9)
*Streptococci*, *other*	156 (8.5)
*Enterococcus faecalis*	44 (2.4)
Monomicrobial Gram-negative	
*Escherichia coli*	633 (34.3)
*Klebsiella spp*.	102 (5.5)
*Pseudomonas aeruginosa*	49 (2.7)
*Other*	145 (7.9)
Polymicrobial	185 (10.0)
**Admitted to**	
Surgical unit	208 (11.3)
Medical unit	1336 (72.5)
Oncology/haematology unit	217 (11.8)
Intensive care unit	79 (4.3)
Unknown	4 (0.2)
**Sepsis severity**	
No sepsis	108 (5.9)
Possible sepsis	237 (12.9)
Sepsis	415 (22.5)
Severe sepsis or septic shock	977 (53.0)
Organ dysfunction without sepsis	107 (5.8)

### Serum albumin levels with respect to the population characteristics

PA levels ranged from 11 to 53 g/L. Almost half of the patients (897 [48.6%]) had hypoalbuminaemia (<35 g/L) of whom 158 (8.6%) had <25 g/L.

PA levels tended to decline with increasing frailty of the patients, especially for increasing severity in the sepsis groups (Table 2). Among the sepsis severity groups, a trend test corroborated the significant p-value by showing decreasing PA levels with an increase in sepsis severity.

**Table 2:** Mean plasma albumin levels on the bacteraemia date for population characteristic groups among adult community-acquired bacteraemia patients (n = 1844).

Population characteristic groups	Mean serum albumin level (g/L)	p-value^1^
**All patients**	34.0	
**Gender**		0.10
Males	34.1	
Females	33.8	
**Age (years)**		0.002
15–64	34.6	A
65–79	33.4	B
≥ 80	33.8	Ab
**Charlson comorbidity points**		<0.0001
0	35.6	A
1	33.7	B
≥ 2	33.4	B
**Bacterial groups**		0.01
Monomicrobial Gram-positive	34.4	A
Monomicrobial Gram-negative	33.8	Ac
Polymicrobial	32.5	Bc
**Sepsis severity**		<0.0001
No sepsis	34.8	A
Possibly sepsis	35.6	A
Sepsis	36.4	a
Severe sepsis or septic shock	32.8	b
Organ dysfunction without sepsis	30.4	c

^1^ For significant p-values, different characters denote significant differences in pairwise comparisons. Distinct letters indicate significant differences within groups defined by population characteristics.

### Kaplan-Meier survival curves

**Fig 1:**
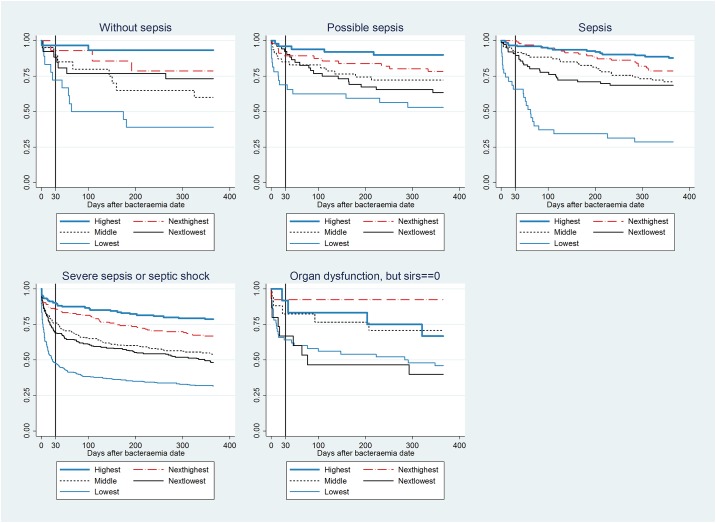
Kaplan-Meier survival curves for five sepsis-groups in the 0–365-day period. For each sepsis group, a separate curve is illustrated for each quintile of the serum albumin level.

Mortality was positively associated with sepsis severity and within all severity groups mortality incidence negatively correlated with PA levels (Fig 1).

The survival curves were non-parallel in the 0–30-day period. Beyond 30 days, the survival curves for the less severe sepsis groups (no sepsis, possible sepsis, and sepsis) continued to have different curve-slopes, whereas the curves became nearly parallel for the groups with severe sepsis groups (severe sepsis/septic shock, organ dysfunction without sepsis).

### Logistic regression analyses

For the 0–30-day mortality rates, ORs did not change appreciably across the P-, PI-, and PIRO-models (Table 3). Irrespective of the model, ORs mainly declined with comorbidity and sepsis either with or without addition of PA level. The ORs of the PA level did not change significantly between the three models, so that a decrease in PA levels by 1 g/L consistently predicted a 13–14% higher mortality incidence.

**Table 3:** Logistic regression analyses for the 0–30-day mortality.

Factor	P^1^	P^1^+albumin	PI^2^	PI^2^+albumin	PIRO^3^	PIRO^3^+albumin
Age (years)	1.03 (1.02–1.04)^4^	1.03 (1.03–1.04)	1.03 (1.02–1.04)	1.04 (1.03–1.05)	1.03 (1.02–1.04)	1.04 (1.03–1.05)
Females	1 (reference)	1 (reference)	1 (reference)	1 (reference)	1 (reference)	1 (reference)
Males	1.38 (1.09–1.74)	1.53 (1.19–1.97)	1.37 (1.08–1.74)	1.52 (1.18–1.96)	1.28 (1.00–1.63)	1.44 (1.11–1.87)
Charlson 0	1 (reference)	1 (reference)	1 (reference)	1 (reference)	1 (reference)	1 (reference)
Charlson 1	1.35 (0.87–2.07)	1.07 (0.68–1.70)	1.33 (0.87–2.05)	1.05 (0.66–1.67)	1.29 (0.83–2.00)	1.03 (0.65–1.66)
Charlson 2	2.29 (1.65–3.20)	1.91 (1.34–2.73)	2.31 (1.65–3.22)	1.96 (1.37–2.80)	2.29 (1.63–3.22)	2.03 (1.41–2.92)
Gr+, mono	-	-	1 (reference)	1 (reference)	1 (reference)	1 (reference)
Gr-, mono	-	-	0.77 (0.60–0.98)	0.68 (0.52–0.88)	0.80 (0.62–1.03)	0.69 (0.53–0.90)
Others	-	-	1.25 (0.74–2.11)	1.00 (0.57–1.76)	1.33 (0.77–2.30)	1.08 (0.60–1.93)
No sepsis	-	-	-	-	1 (reference)	1 (reference)
Possibly sepsis	-	-	-	-	1.06 (0.51–2.20)	1.31 (0.61–2.83)
Sepsis	-	-	-	-	0.61 (0.30–1.25)	0.82 (0.38–1.73)
Severe sepsis	-	-	-	-	3.03 (1.61–5.67)	2.83 (1.45–5.49)
Organ dysf.	-	-	-	-	2.58 (1.21–5.50)	1.62 (0.72–3.63)
Albumin	-	0.86 (0.84–0.88)	-	0.86 (0.84–0.88)	-	0.87 (0.85–0.89)

^1^Predisposition;

^2^predisposition and insult/infection;

^3^predisposition, insult/infection, and response/organ dysfunction;

^4^odds ratio (95% confidence interval).

Sepsis severity was not a significant predictor of mortality during the 31–365-day period (Table 4). In contrast to the 0–30-day mortality, there were only minimal changes in ORs when PA level was added to the models. As for the 0–30-day mortality, the ORs of PA level remained fairly constant regardless of the model. The OR of 0.93 meant that a decrease in PA level by 1 g/L predicted a 7% increase in mortality, i.e., half as much of the PA predicting capacity during the 0–30-day period. The OR was 0.89 for the three less severe conditions, but comprised 0.95 for the two groups with more severe pathologies (data not shown), which corroborated the higher diversions for the former as depicted in Fig 1.

**Table 4:** Logistic regression analyses for the 31–365-day mortality.

Factor	P^1^	P^1^+albumin	PI^2^	PI^2^+albumin	PIRO^3^	PIRO^3^+albumin
Age (years)	1.03 (1.02–1.04)^4^	1.03 (1.02–1.04)	1.03 (1.02–1.04)	1.03 (1.02–1.04)	1.03 (1.02–1.04)	1.03 (1.02–1.03)
Females	1 (reference)	1 (reference)	1 (reference)	1 (reference)	1 (reference)	1 (reference)
Males	1.21 (0.93–1.57)	1.26 (0.96–1.64)	1.21 (0.93–1.57)	1.26 (0.96–1.65)	1.18 (0.91–1.54)	1.24 (0.95–1.62)
Charlson 0	1 (reference)	1 (reference)	1 (reference)	1 (reference)	1 (reference)	1 (reference)
Charlson 1	2.32 (1.32–4.07)	2.16 (1.22–3.81)	2.29 (1.30–4.02)	2.13 (1.21–3.76)	2.29 (1.30–4.03)	2.14 (1.21–3.78)
Charlson 2	7.00 (4.42–11.05)	6.61 (4.16–10.50)	6.90 (4.37–10.92)	6.51 (4.10–10.35)	6.93 (4.38–10.97)	6.57 (4.13–10.45)
Gr+, mono	-	-	1 (reference)	1 (reference)	1 (reference)	1 (reference)
Gr-, mono	-	-	1.13 (0.85–1.49)	1.11 (0.84–1.48)	1.15 (0.87–1.52)	1.13 (0.85–1.50)
Others	-	-	1.82 (0.99–3.36)	1.81 (0.97–3.36)	1.91 (1.03–3.54)	1.89 (1.02–3.54)
No sepsis	-	-	-	-	1 (reference)	1 (reference)
Possibly sepsis	-	-	-	-	0.74 (0.38–1.41)	0.80 (0.41–1.56)
Sepsis	-	-	-	-	0.96 (0.53–1.73)	1.12 (0.61–2.07)
Severe sepsis	-	-	-	-	1.25 (0.71–2.19)	1.24 (0.69–2.20)
Organ dysf.	-	-	-	-	1.03 (0.48–2.20)	0.83 (0.38–1.81)
Albumin	-	0.93 (0.91–0.95)	-	0.93 (0.91–0.95)	-	0.93 (0.90–0.95)

^1^Predisposition;

^2^predisposition and insult/infection;

^3^predisposition, insult/infection, and response/organ dysfunction;

^4^odds ratio (95% confidence interval).

### The area under the ROC curves

For the 0–30-day mortality, the PA level contributed more to the AUC value (0.78 for the P-model with PA) than both bacterial and sepsis group factors (0.73 for the PIRO-model without PA) (Table 5). For the models with PA, there was less impact on the AUC values when the sepsis-groups were introduced. Irrespective of the model, the largest relative changes in AUC values, ranging from 11% to 18%, were seen when PA level was added. All models with PA level reported an AUC value close to 0.80, which is often considered clinically satisfactory.

**Table 5:** Values of the area under the ROC curve (95% confidence interval) (white cells) and % change for different models (grey cells).

	0–30 day mortality			31–365 day mortality		
Model	Without albumin	% increase	With albumin	Without albumin	% increase	With albumin
P-model^1^	0.66 (0.64–0.69)	18.2	0.78 (0.76–0.81)	0.71 (0.68–0.74)	4.2	0.74 (0.71–0.77)
% increase	1.5		1.3	1.4		1.4
PI-model^2^	0.67 (0.64–0.70)	17.9	0.79 (0.76–0.81)	0.72 (0.69–0.74)	4.2	0.75 (0.72–0.77)
% increase	9.0		2.5	0.0		0
PIRO-model^3^	0.73 (0.70–0.76)	11.0	0.81 (0.79–0.83)	0.72 (0.70–0.75)	4.2	0.75 (0.72–0.78)

^1^Predisposition (age, gender, comorbidity);

^2^predisposition and insult/infection (age, gender, comorbidity, main bacterial groups);

^3^predisposition, insult/infection, and response/organ dysfunction (age, gender, comorbidity, main bacterial groups, sepsis severity groups)

For the 31–365-day mortality, the AUC value in the P-model that included the PA level (0.74) was larger than the AUC value of the PIRO-model without PA (0.72). In contrast to the case of the 0–30-day mortality, the sepsis-groups contributed very little to the AUC value. Although the PA level still contributed more to the AUC values than any other amended variable, its impact was much smaller than that for the 0–30-day mortality.

### Patients with serum albumin measurements 8–30 days before the bacteraemia

Among the 1844 patients considered, 422 (23.3%) had one or more PA measurements in the period from 8 to 30 days before the bacteraemia date. The average number of days from the latest 8–30-day PA measurement to the bacteraemia date was 15.4 ± 6.6 (data not shown). The average PA level during the latest measurement was 35.3 ± 5.8 g/L (data not shown), i.e., 1.3 g/L higher than the level determined on the bacteraemia date (Table 1).

**Fig 2:**
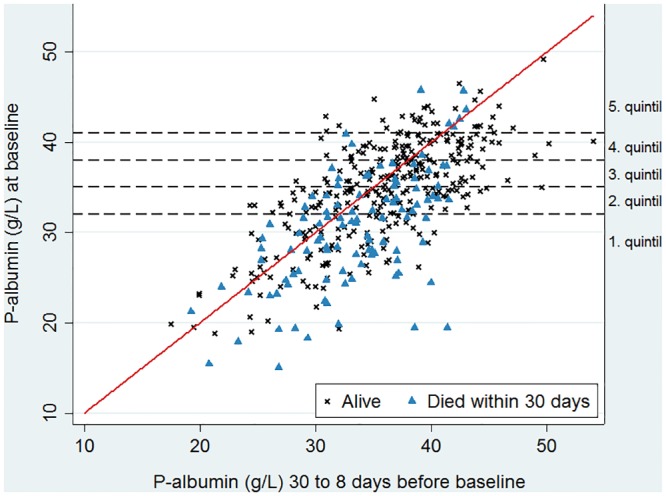
Scatter plot of plasma albumin (PA) levels for 422 patients who had one or more PA measurements in the period from 8–30 days before the bacteraemia date. The x-axis depicts the last measured albumin level in the 8–30-day period and the y-axis depicts the albumin level on the bacteraemia date. Dots below the diagonal line represent patients with a decline in the albumin level between the last measurement in the 8–30-day period and the measurement on the bacteraemia date, whereas dots above that line represent a corresponding increase in the PA level. Data for patients, who remained alive 30 days after the bacteraemia date, are represented by crosses, while triangles indicate data for patients who died within 0–30 days after the bacteraemia date.

To visualise the data from those 422 patients, we plotted the latest measured PA level during the 8–30-day period on the x-axis and the baseline PA level, determined on the bacteraemia day, on the y-axis (Fig 2). The diagonal line in Fig 2 represents no change in PA levels. Dots, representing individual patients, below that line indicate that there was a decrease in the PA level from the 8–30-day period to the bacteraemia date, whereas dots above that line, in contrast, point to an increase in the PA level in that period. Most patients had a decrease in their PA level. Moreover, the distribution of individual cases in Fig 2 was clearly asymmetrical: in the lower quintiles, relatively more patients had decreased PA levels as compared to PA levels in the higher quintiles.

A visual inspection of the graph suggests that a higher proportion of patients died within 30 days of the bacteraemia episode, when the baseline PA levels were low. Only for patients with very high albumin levels 8–30 days before the bacteraemia date (data in the 5th quintile), it was not visually apparent that a higher proportion of them died within 30 days after the bacteraemia episode when their PA levels determined on the bacteraemia date were low.

## Discussion

We found that the PA level was a strong prognostic mortality predictor for adult community-acquired bacteraemia patients, especially for the mortality in the short period after the bacteraemia episode. To the best of our knowledge, this is the first study that has evaluated PA levels in relation to both chronic and acute factors in bacteraemia patients by a PIRO model. This enabled quantification of the PA level prognostic capacity as compared to the influence of chronic and acute comorbidities, both of which are common in bacteraemic patients.

Bacteraemia is a serious condition with a 30-day mortality rate of 15–30% [19]. It is difficult to assess the relative impact of the infection *per se* and of other conditions on cases with poor outcomes. In order to better differentiate between predisposition factors (such as old age and comorbidity) and acute factors related to the septic episode, the PIRO (Predisposition, Insult/Infection, Response, Organ dysfunction) model was introduced a decade ago [17]. The PIRO model has proved valid in studies of sepsis patients, as there were highly significant associations between mortality rates and PIRO scores [20,21]. The use of various biomarkers in the PIRO models has been advocated previously [20,22], but so far only parameters related to white blood cells (e.g., SIRS criteria) have been included. Other models, such as the SOFA (Sepsis-related Organ Failure Assessment) [23] or the APACHE (Acute Physiology, Age, Chronic Health Evaluation) score [24], have also shown high discrimination in mortality prognosis, though they have not necessarily been superior to the PIRO score [25]. Interestingly, the inclusion of the PA level in APACHE improved its mortality discrimination, after which APACHE II was renamed APACHE III [24]. In this study, we did not opt for the “perfect” prediction model, but rather for an explanatory model [26], which could more specifically assess the role of albumin. Hence, we chose to apply a stepwise PIRO model approach (P, PI, and PIRO) and incorporated the baseline PA level in each step to deduce its impact on predisposition factors representing both chronic conditions and acute factors related to the inflammatory response in bacteraemia.

We found that a lower baseline PA level independently predicted higher mortality during the 0–30 and 31–365-day periods after the bacteraemia episode. The predicting capacity was stronger for mortality in the 0–30-day period. Moreover, the PA level exhibited collinearity with the sepsis severity, which emphasises the reliability of the baseline PA level as a predictor of mortality shortly after bacteraemia. Our results obtained after studies of data from a relatively large patient cohort, suggest that a single PA measurement on the bacteraemia date can be used as a prognostic factor for mortality in the short term after bacteraemia with better predictability than the sepsis severity. These findings were corroborated by the weaker effect of the PA level on the 31–365-day mortality, on which sepsis severity had no prognostic impact. Finally, lower baseline PA levels for most hypoalbuminaemic patients compared to those measured within 8–30-day period before the bacteraemia also indicated that hypoalbuminaemia primarily reflected acute, rather than chronic, conditions, especially given albumin’s long half-life of 20 days [7].

Our finding of hypoalbuminaemia as an independent mortality predictor is not novel. Numerous studies comprising different patient groups, settings, and covariates have unanimously confirmed that hypoalbuminaemia predicts a poor outcome [8,27] and this was also applied to several studies that focused on bacteraemic patients [28–32].

Only a few studies, however, have attempted to assess whether the poor prognosis of patients with hypoalbuminaemia is mainly related to chronic or acute conditions, which are both prognostically relevant in bacteraemia patients. One study that comprised Swedish elderly patients with community-acquired pneumonia [33] and two studies including intensive care unit patients [34,35] supported our view that hypoalbuminaemia mainly reflected an acute inflammatory response. Our results are in line with results reported in those studies. The relation of the PA level to acute conditions was further supported by the decline in the baseline PA level compared to the latest PA level in the period between 8–30 days before the bacteraemia episode, especially amongst hypoalbuminaemic patients. We selected the 8–30 day period assuming that acute conditions related to the bacteraemia, or a possible prior focal infection (such as pneumonia or urocystitis), would play a minor role (see Fig 2). The role of hypoalbuminaemia in acute conditions is further indicated by its inverse correlation with several acute phase biomarkers [1,2,4,36–41]. In contrast to the sepsis variables, the PA level also predicted mortality beyond day 30, especially in the groups with less severe disease (no sepsis, possible sepsis, sepsis). This indicated that the PA level at the time of blood culture to some extent also reflected the presence of chronic conditions having an impact on survival. In addition, we detected an inverse correlation between the PA and serum CRP levels (R = -0.21, *p* < 10^−5^, data not shown) in our data; however, we did not include CRP in our models due to the high collinearity between CRP and the sepsis severity parameters detected in our earlier study for virtually the same study population [9].

Our study was population-based and included a high number of patients with well-defined bacteraemia for whom complete short- and long-term follow-ups were possible. The study, however, had some limitations. Firstly, it was not a prospective study and we only had two or more PA measurements for about 23% of the study population. For these patients, the age and PA levels did not differ from the remaining 77%, but this group had more comorbidity (data not shown). They also had higher mortality levels in the 31–365-day period, but this difference failed to achieve statistical significance after adjusting for comorbidity. We selected the last PA measurement at least 8 days before the baseline PA measurement to represent a period in which the impact of an acute event on the PA level was probably minimal. Secondly, we had no data on the nutritional status, which is traditionally believed to be related to hypoalbuminaemia [7,42]. Newer studies have, however, consistently reported a lack of association between the albumin level and nutrition markers, such as BMI or triceps skinfold thickness [43–45], indicating that albumin is not a sensitive nutritional marker [2]. Thirdly, only 1844 of 2784 patients (66.2%) had their PA level measured on the bacteraemia date, which compromised the population-based design. The 940 patients without a PA measurement were older, had less comorbidity, and were less likely to have sepsis. Fourthly, other prognostic models than PIRO, e.g. SOFA [23] or APACHE III [24] could have been applied, but this was not possible due to lack of data in our retrospective study. There is much discussion on which model is the best to predict morbidity or mortality [26,46], which probably also differs between patient groups and settings [25]. In this study, we preferred an explanatory model in which we focused on one predictor (the PA level), in contrast to a prediction model that assesses all relevant predictors which together render the best prognostic discrimination [26]. We found that the PIRO model, due to its stepwise approach and ability to distinguish between chronic and acute factors, was a suitable hypothesis-generating explanatory model [17]. Finally, due to missing data it was unclear if some of the patients had sepsis or not. We reiterated all the analyses with these patients as either having no sepsis or sepsis, but that manipulation did not change the results significantly (data not shown).

In conclusion, the baseline PA level was a strong prognostic mortality predictor for adult community-acquired bacteraemia patients, especially for mortality shortly after the bacteraemic episode. Its negative correlation with mortality rates during day 0–30 and 31–365 did not change after an adjustment with chronic or acute factors. Moreover, for the short-term mortality, PA level was a stronger predictor than sepsis severity indices based on the commonly used SIRS criteria and organ dysfunction parameters [12]. This retrospective study indicates that hypoalbuminaemia was mainly caused by factors related to the acute response *per se*. Prospective studies with longitudinal PA measurements are necessary to further elaborate dynamics of albumin level changes in relation to acute conditions. However, this can only be achieved in patients, which are followed routinely due to other diseases.
